# Infertility as a Consequence of Spermagglutinating *Staphylococcus aureus* Colonization in Genital Tract of Female Mice

**DOI:** 10.1371/journal.pone.0052325

**Published:** 2012-12-18

**Authors:** Siftjit Kaur, Vijay Prabha

**Affiliations:** Department of Microbiology, Panjab University, Chandigarh, India; Cincinnati Childrens Hospital Medical Center, United States of America

## Abstract

Various studies have shown *Staphylococcus aureus* to be one of the most prevalent organism in male and female genital tract but most practitioners dismiss it as mere contamination which is assumed to be of no significance. However, it is now suggested that the presence of this organism should not be ignored, as incubation of spermatozoa with *S. aureus* results in reduced sperm motility. Although *S. aureus* has been reported to cause immobilization of spermatozoa, however, its role in infertility has yet to be elucidated. The present study was designed to establish a spermagglutinating strain of *S. aureus* isolated from the cervix of a woman with unexplained infertility, in mouse and evaluate its effect on fertility outcome. Female Balb/c mice were inoculated intravaginally with different doses of *S. aureus* (10^4^, 10^6^ or 10^8^cfu/20 µl) for 10 consecutive days. Microbial colonization monitored every 3^rd^ day by vaginal cultures, revealed that strain could efficiently colonize mouse vagina. Mating on day 12, with proven breeder males led to 100% decrease in fertility as compared to control. Even a single dose of 10^6^ or 10^8^cfu could lead to vaginal colonization which persisted for 10 days followed by gradual clearing till 21 days, vaginal cultures were negative thereafter. Female mice mated on day 7 (culture positive), were rendered infertile, however, the mice mated on day 22 (culture negative), retained fertility and delivered pups indicating its role in provoking infertility. Further, except infertility, no other clinical manifestation could be seen apparently or histologically. However, when a non-spermagglutinating/immobilizing standard strain of *S. aureus* MTCC6625 was inoculated intravaginally at 10^8^cfu for 10 days followed by mating on day 12, fertility was observed in all the female mice. This supports the hypothesis that infertility observed in the former groups was as a result of colonization with spermagglutinating strain of *S. aureus.*

## Introduction

Controversies exist in literature regarding the role of bacterial infection in infertility. Barring the role of a few bacteria such as Chlamydia whose impact on fertility has been well established, the role of other bacteria in infertility is debatable [1]. Though, many microorganisms have been isolated from seminal fluid samples of infertile patients, but it has not been clearly established as to whether any of the organisms isolated actually causes infertility. Momoh et al. [2] reported that *Staphylococcus aureus* is one of the dominant microorganism with a prevalence rate of 38.7% from high vaginal swab and endocervical swabs, respectively and a prevalence of 75% among the bacterial strains from semen cultures of infertile couples.

Various bacteria such as *Escherichia coli*, *Ureaplasma urealyticum*, *Mycoplasma hominis* and *S. aureus* have been known to impede sperm motility *in vitro* [3]–[6]. *In vitro* studies have shown that *E. coli* and *M. hominis* also attach to human spermatozoa leading to morphological changes and decrease in the fertilization potential of sperm. What role do these bacteria play *in vivo* has not been explored and neither has their presence linked with infertility. The *S. aureus* strain used in the present study was isolated from the cervix of female with unexplained infertility. The strain was shown to impede sperm motility by causing spermagglutination and could also adhere to human spermatozoa [7], [8]. The strain showed interesting results *in vitro*, but whether it also had the potential to cause infertility was the subject of the present study. Therefore, we established a spermagglutinating strain of *S. aureus* in the vaginal tracts of female Balb/c mice and studied its effect on fertility outcome.

**Table 1:** Effect of repeated intravaginal inoculations of spermagglutinating strain of *S. aureus* on fertility outcome in mice.

Dose instilled as 10 consecutive inoculations (cfu/20µl)	No. of treated mice	Fertility outcome
Control (PBS)	3	3/3 (100%)
10^4^	3	0/3 (0%)
10^6^	3	0/3 (0%)
10^8^	3	0/3 (0%)

Experiment was repeated twice and identical results were obtained.

## Materials and Methods

### Microorganism

The strain of *S. aureus* used in the present study was isolated earlier in our laboratory from the cervix of a woman suffering from unexplained infertility. This strain showed remarkable spermagglutinating property *in vitro* (Figure S1) and could also adhere to human spermatozoa [7], [8]. Additionally, a standard strain of *S. aureus* MTCC6625 was procured from Microbial Type Culture Collection (MTCC), Institute of Microbial Technology (IMTECH), Sector-39, Chandigarh, India. This strain did not show any effect on motility of human spermatozoa *in vitro* and served as negative control (Figure S2). Both the strains were maintained on brain heart infusion (BHI) agar and were stored as glycerol stocks at −80°C.

**Figure 1:**
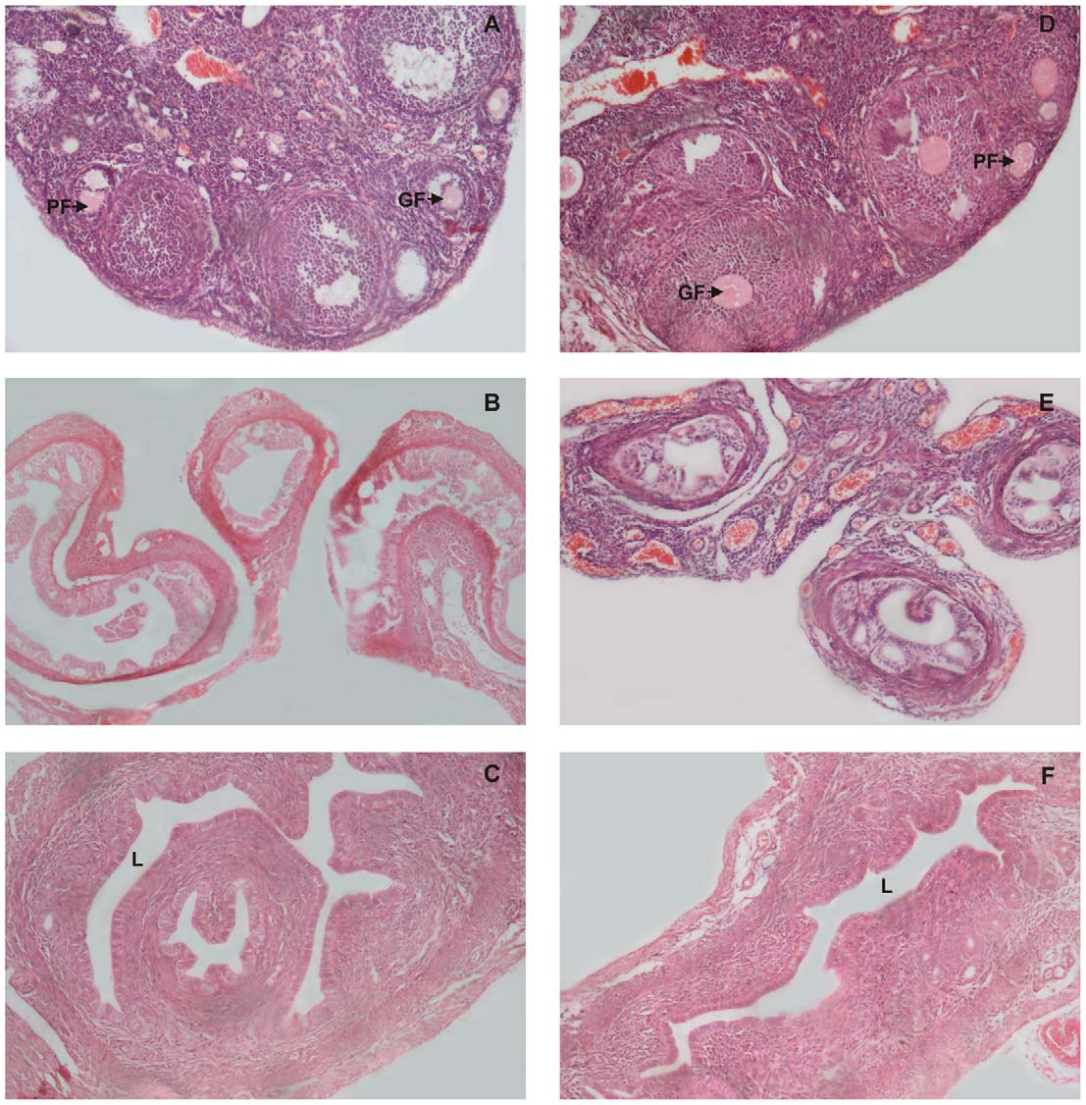
Histological examination of reproductive organs on day 12 after intravaginal administration of PBS (Control) or spermagglutinating strain of *S. aureus* for 10 consecutive days. Hematoxylin and eosin stained sections and light micrographs of the ovaries, fallopian tubes and uterus of mice, 12 days after inoculation with *S. aureus* (D–F) compared with control sections (A–C). Ovaries, fallopian tubes and uterus show normal histology in both the groups. Graaffian and primodial follicles are prominently seen in ovaries of both the groups (PF and GF). Original magnification 100X.

**Figure 2:**
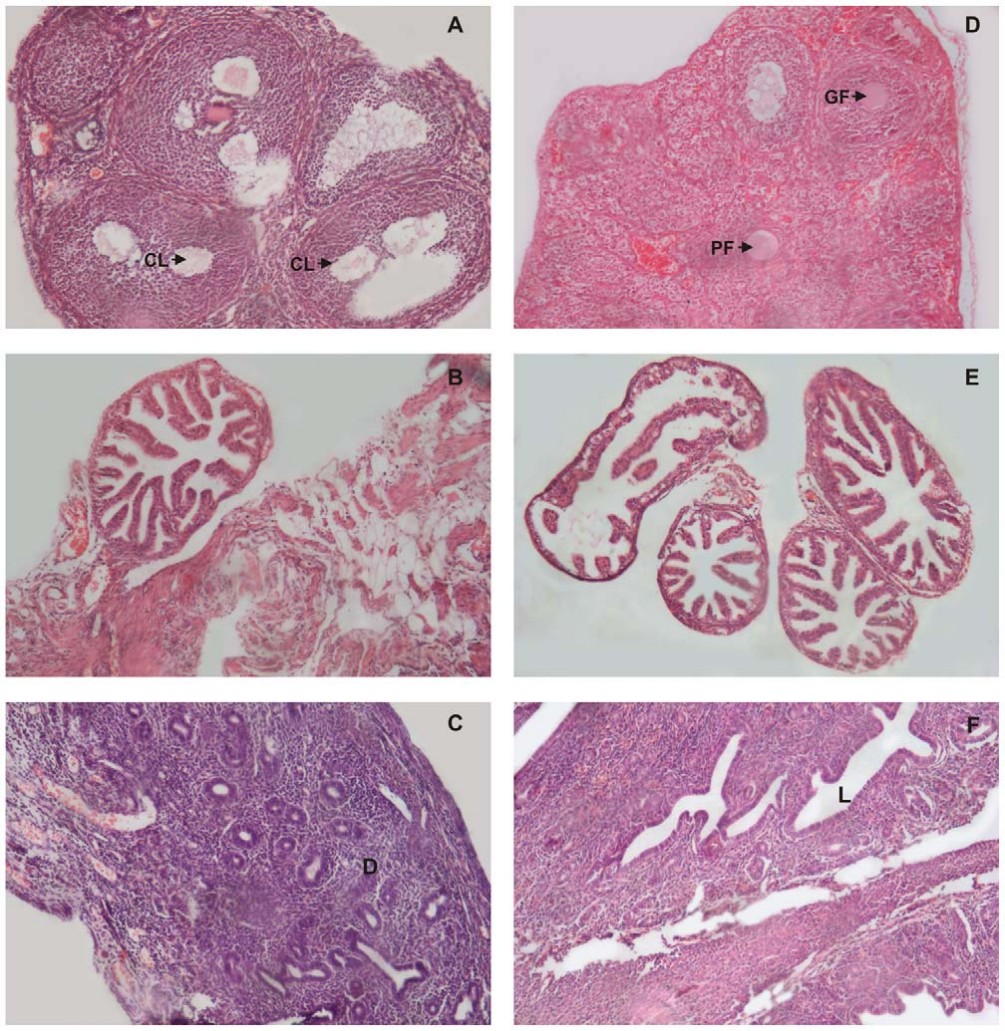
Histological examination of reproductive organs on day 19 after intravaginal administration of PBS (Control) or spermagglutinating strain of *S. aureus* for 10 consecutive days. Light microscopic images of sections of reproductive organs on day 19 of control mice showing follicles and corpus luteum (CL) in the ovary and proliferation of endometrium in the uterus characteristic of pregnancy (A & C), in contrast to sections from *S. aureus* treated mice showing no such pregnancy related changes (D & F). Fallopian tubes from control and treated mice were indistinguishable (B & E). Original magnification 100X.

**Figure 3:**
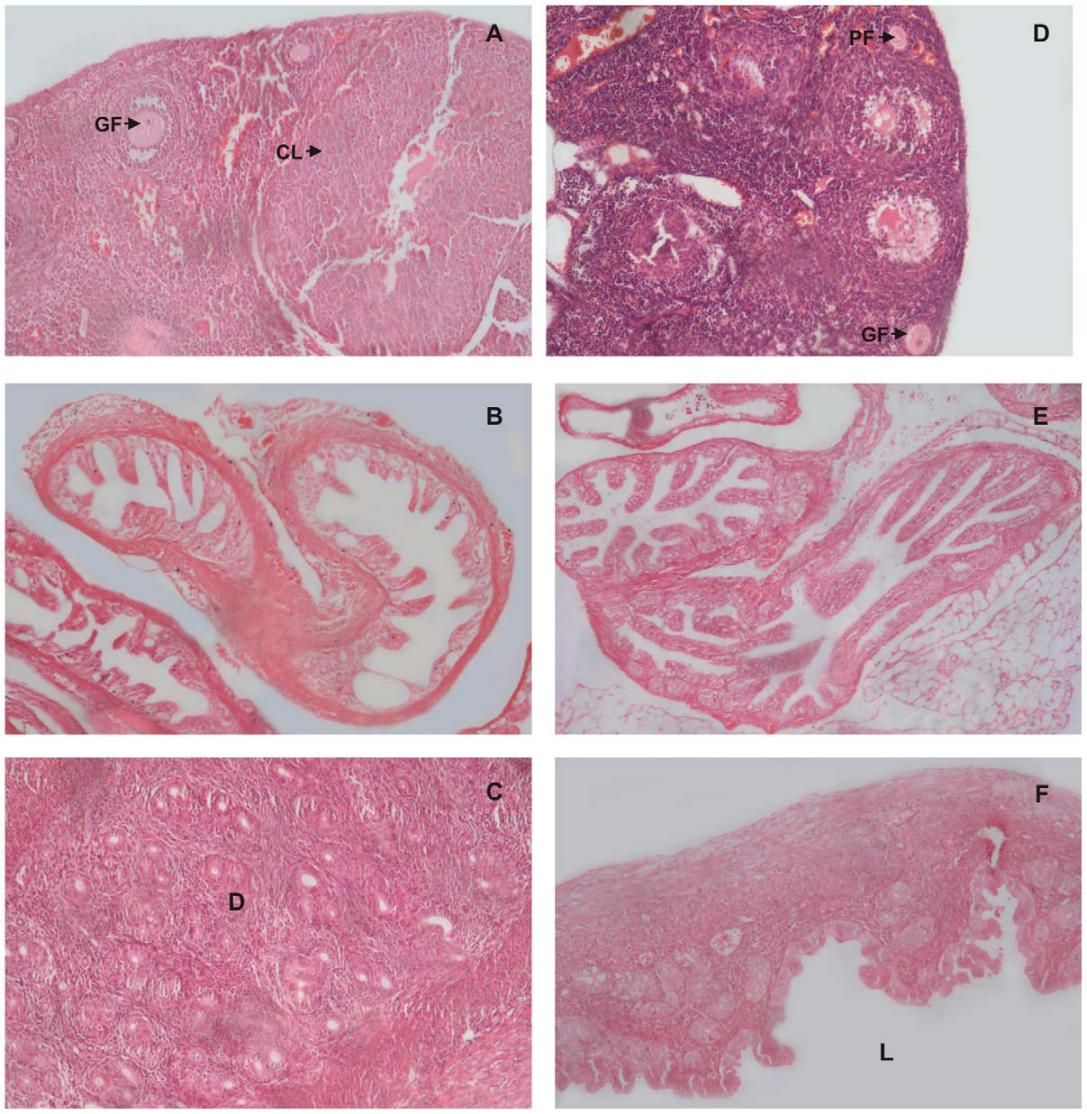
Histological examination of reproductive organs on day 26 after intravaginal administration of PBS (Control) or spermagglutinating strain of *S. aureus* for 10 consecutive days. Histology of the female reproductive tract on day 26 in mouse administered with PBS only (A, B, C) or with *S. aureus* (D, E, F) for 10 consecutive days. Control ovary (A) shows the persistence of corpus luteum (CL) and uterus (C) shows extensive proliferation of endometrium with formation of decidua (D, indicated by arrow). In *S. aureus* inoculated mice there is absence of corpus luteum in the ovary (D) and decidua in the uterus (E). Fallopian tubes in both the groups show normal histology (B & F).

### Animals

Sexually mature, 5–6 week old male (25±2 g) and 4–5 week old female (22±2 g) Balb/c mice were used in the present study. The animals were housed in polypropylene cages and kept in the animal room of the Department of Microbiology, Panjab University, Chandigarh. The animals were maintained in laboratory conditions (12∶12, dark:light cycle) and fed with standard pellet diet and water *ad libitum*. The experimental protocols were approved by the Institutional Animal Ethics Committee of the Panjab University, Chandigarh, India vide letter no. 1500/CAH dated 30.09.09 and were performed in accordance with the guidelines of the Committee for the Purpose of Control and Supervision of Experiments on Animals (CPCSEA).

**Table 2:** Effect of single intravaginal inoculation of spermagglutinating strain of *S. aureus* on fertility outcome in mice.

	Set I		Set II	
	Mating on day 7 post inoculation (culture positive mice)		Mating on day 22 post inoculation (culture negative mice)	
No. of *S. aureus* (cfu/20µl)	No. of mice tested	Fertility outcome	No. of mice tested	Fertility outcome
Control (PBS)	3	3/3 (100%)	3	3/3 (100%)
10^6^	3	0/3 (0%)	3	3/3 (100%)
10^8^	3	0/3 (0%)	3	3/3 (100%)

### Screening of animals

First, we screened the animals for the micro-organisms that naturally inhabit the Balb/c mouse vagina. The microbiota of the mice was studied from vaginal samples taken with sterile cotton swabs moistened with physiological saline. Swabs were cultured at 37°C on plates of brain heart infusion (BHI) agar and mannitol salt agar (MSA).

**Figure 4:**
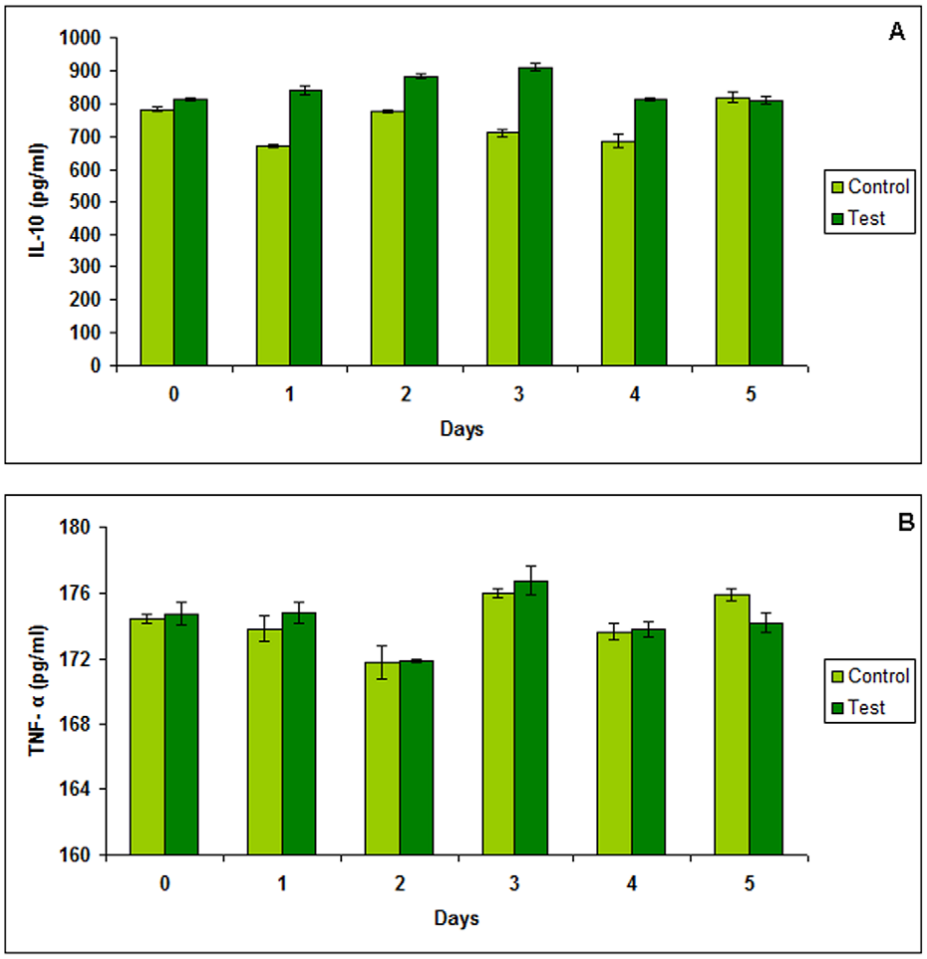
**Estimation of IL-10 and TNF-α levels in mouse vaginal homogenates.** Determination of (A) IL-10 and (B) TNF-α levels in mouse vaginal homogenates collected at 24 h intervals from days 0, 1, 2, 3, 4 and 5, during vaginal administrations of 10^8^cfu of *S. aureus.* Data represents mean of three observations. Results are expressed as mean ± S.D.

Strains growing on BHI agar and MSA plates were further checked for spermagglutinating/immobilizing properties. All the mice harbouring either *S. aureus* or any other bacteria with spermagglutinating/immobilizing property were excluded from the study.

**Figure 5:**
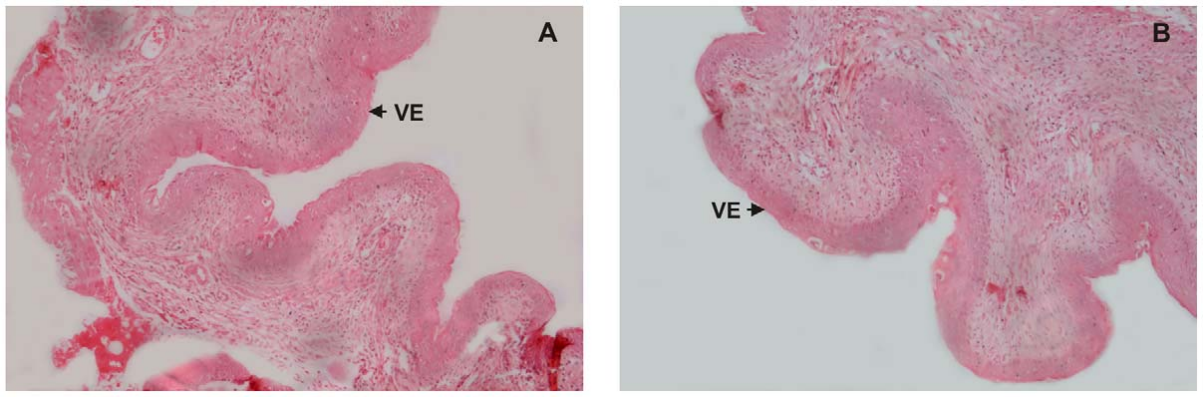
Histopathological examination of vaginal tissues. Hematoxylin and eosin stained sections and light micrographs of mouse vagina after 10 days of daily treatment with 10^8^cfu of spermagglutinating strain of *S. aureus* or PBS. Normal vaginal epithelium (VE) is observed in the control and the test samples. No histopathological changes were observed in the vaginal sections (ulceration, edema, or leukocyte infiltration) of the test (A) or in the representative control receiving PBS (B). Original magnification 100X.

### Effect of multiple doses of spermagglutinating strain S. aureus on fertility outcome

#### Preparation of inoculums

*S. aureus* was cultivated in BHI broth at 37°C for 24 h. The cell culture was centrifuged at 10,000 g for 20 min and washed twice with PBS (50 mM, pH 7.2). The cells were resuspended in the same buffer so as to get 10^4^, 10^6^ and 10^8^cfu/20 µl. Similarly, the non-agglutinating/immobilizing standard strain was grown in BHI broth and was adjusted to 10^8^cfu/20 µl of PBS.

#### Intravaginal inoculation

To ascertain the dose needed to evaluate colonization and induce infertility, three groups of female mice without under any anesthesia were inoculated intravaginally with 10^4^, 10^6^ or 10^8^cfu of *S. aureus* per mouse in 20 µl PBS for 10 consecutive days. As control, mice were inoculated intravaginally either with 20 µl of PBS alone or 10^8^cfu of non-agglutinating/immobilizing standard strain of *S. aureus* for 10 consecutive days.

Vaginal swabs were taken every 3^rd^ day so as to monitor vaginal colonization. The reisolated bacteria from the vaginal swabs were further confirmed as *S. aureus* by culture characteristics and biochemical tests and were also checked for spermagglutinating activity *in vitro*.

On day 12, these female mice were allowed to mate with breeder male mice in the ratio 2∶1, to check the effect on fertility outcome. Upon mating, next day the females were separated and mating was confirmed by observing the presence of vaginal plug in all the female mice. For the entire experimental period, animals were examined for behavioral changes and vaginal discharges or bleeding. After mating, mice were checked for fertility related changes i.e. weight gain and abdominal distension. Each group included three mice and the experiment was done twice amounting to total number of animals used in this study 30.

### Histological Studies

In parallel, 30 other mice, with 6 mice in each group were similarly inoculated and mated. Two mice from each group were killed on day 12, 19 and 26 for histological analyses. The reproductive organs (ovaries, fallopian tubes and uterus) were collected and histological examination was carried out by bright field microscopy. The organs were removed, fixed in 10% formaldehyde for 24 h and then embedded in paraffin according to standard histological methods. Serial paraffin sections of 4 µm were stained with hematoxylin-eosin and observed at 40X.

### Effect of single dose on fertility outcome

As multiple intravaginal inoculations of spermagglutinating strain of *S. aureus* in female mice led to compromised fertility, interest was developed to determine if a single inoculation could also lead to similar outcome.

For this, three groups of female mice with six mice in each group (24 mice) were inoculated intravaginally with a single dose of 10^4^, 10^6^ and 10^8^cfu/20 µl of *S. aureus*. As control, six mice were inoculated intravaginally with 20 µl of PBS alone. As mice inoculated with 10^4^cfu/20 µl did not show colonization of *S. aureus* for more than 6 days, therefore, further studies were not carried out with this group. Rest of the two groups were divided into two sets with three mice per group in each set.

In the first set, mice were mated in the presence of organism on day 7 i.e. within the period when the vaginal cultures were positive for *S. aureus*. In the second set, the female mice were mated upon the complete clearance of the organism from the vaginal tract, on day 22 i.e. when the mice were culture negative for *S. aureus*.

Upon mating, next day the females were separated and mating was confirmed by observing the presence of vaginal plug. Pregnancy was determined by weight gain and by observing abdominal distension.

### Effect of repeated intravaginal inoculation of S. aureus on tissue histology and cytokine levels

As no apparent clinical symptoms were observed except infertility, subsequent studies were done to examine the effect of 10 day intravaginal inoculation of 10^8^cfu of *S. aureus* on vaginal histology and changes (if any) in the immunological markers. 21 mice were inoculated with 10^8^cfu of *S. aureus* for 10 consecutive days (test group) and 21 mice were administered with 20 µl of PBS alone (control group). Out of 42, 36 mice were sacrificed with three mice per group on day 0, 1, 2, 3, 4 and 5 by cervical dislocation and vaginal tissues were collected. The vaginal tissues were suspended in normal saline (0.85%) and homogenized with a Teflon pestle. The vaginal homogenates were used for the estimation of IL-10 and TNF-*α* level.

Further, the remaining mice (n = 3, in each group) in both the groups were sacrificed on day 14 and the vaginal tissues were collected and histopathological examination was carried out.

### Cytokine Immunoassays

Mouse-specific IL-10 and TNF-*α* levels in vaginal lavages and vaginal tissue homogenates fluids collected on day 0, 1, 2, 3, 4 and 5 post intravaginal inoculation with *S. aureus* were measured by a solid phase sandwich enzyme linked immunosorbant assay (ELISA). Samples were assayed for immunoreactive murine IL-10 and TNF-*α* using ELISA kits obtained from Raybiotech (Norcross, GA, USA). As preliminary studies, vaginal lavages did not show appreciable levels of both these cytokines, therefore, vaginal homogenates were used for the present work. In all experiments, tissue homogenates from PBS-only controls were used in parallel. All ELISA kits used mouse-specific monoclonal antibodies as capture antibody and prebiotinylated mouse-specific secondary antibody. Bound biotinylated antibody was detected by the addition of Streptavidin-horse radish peroxidase conjugate. Tetramethylbenzidine (TMB) plus hydrogen peroxide was used as substrate and the signal was amplified with 2N sulfuric acid to convert the product from a blue to a yellow colour. Absorbance of the oxidized TMB was read at 450 nm versus a substrate blank using a microplate reader (Bio-Rad, India). Cytokine quantitation was expressed as picograms per milliliter (pg/ml) in tissue homogenate. The standard curve for the assays ranged from 0–5000pg/ml for IL-10 (Figure S3) and 0–1500pg/ml for TNF-*α* (Figure S4). Results are expressed as the mean ± standard deviation (S.D.) of the data obtained from three animals.

### Histopathological Studies

Mice were sacrificed on day 14, vaginal tissues were collected and histopathological examination was carried out by bright field microscopy for inflammatory changes. Vagina were aseptically removed and fixed in 10% formaldehyde and processed as described earlier.

## Results

### Effect of multiple intravaginal inoculations of spermagglutinating strain of S. aureus on fertility outcome in mice

When the female mice were inoculated intravaginally with different doses (10^4^, 10^6^ or 10^8^cfu/20 µl) of *S. aureus* for 10 consecutive days, it was observed that at all the doses bacteria could efficiently colonize mouse vagina. Upon mating on day 12 with proven male breeder mice, a 100% decrease in fertility was observed as compared with control group. All the nine mice (3 per group) failed to conceive as no pregnancy related changes such as weight gain or abdominal distention was observed. This was in contrast to the control group mice which showed consistent weight gain, string of pearls could be palpated by day 14 and mice delivered pups at the end of gestation period i.e. 21 days. Interestingly, the group receiving intravaginal inoculations of 10^8^cfu of non-agglutinating/immobilizing standard *S. aureus* strain also retained fertility as the mice showed pregnancy related changes i.e. consistent weight gain comparable to the control group and delivered pups at the end of gestation period (Table 1).

Histological examination on day 12 (0 day post mating) did not show any significant change in the reproductive organs of test and the control groups (Figure 1 A, B, C, D, E, F). whereas, on day 19 (i.e. day 7, post mating), the element of corpus luteum could be observed in the ovary of control group (Figure 2A), which was consistently preserved in the following ovarian sections on day 26 i.e. day 14 post mating (Figure 3A) indicative of the pregnancy induced luteal phase which persists due to the secretion of progesterone. The uterus also showed pregnancy related changes in the control group. On day 19, there was increase in the thickness of the endometrium followed by extensive proliferation of uterine glands (Figure 2C). By day 26, the deciduas formation could be observed in the uterus (Figure 3C). Similar observations were made in the reproductive organs of mice inoculated with 10^8^cfu non-agglutinating/immobilizing standard strain of *S. aureus.* All these findings relate to pregnancy. However, these changes were absent in the reproductive organs of mice inoculated either with 10^4^, 10^6^ or 10^8^cfu of spermagglutinating strain of *S. aureus* indicating absence of pregnancy (Figure 2D, E, F, 3D, E, F).

### Effect of single intravaginal inoculation of spermagglutinating strain of S. aureus on fertility outcome in mice

Female mice were inoculated with 10^4^, 10^6^ or 10^8^cfu of *S. aureus*, per mouse in 20 µl of PBS. At a dose of 10^4^cfu, colonization with *S. aureus* could not be maintained in the vagina as the organism cleared by day 6. On the other hand, at 10^6^ or 10^8^cfu the organism persisted in the vagina for 10 days. The bacterial counts started decreasing from day 10 and were gradually cleared by day 16 and 21, respectively, the vaginal cultures were negative thereafter. As the significant bacterial load in the vagina was maintained for 10 days for 10^6^ and 10^8^cfu, therefore, mice in set I were mated in the presence of the organism i.e. on day 7 and in set II after complete clearance of the organism i.e. on day 22. Interestingly, all the female mice mated on day 7, were rendered infertile which was in sharp contrast to the set mated on day 22, which retained fertility (Table 2). These mice showed a consistent weight gain, followed by abdominal distension and delivered pups. Further, except infertility, no other clinical manifestation could be seen apparently.

### Effect of repeated intravaginal inoculation of S. aureus on tissue histology and cytokine levels

As no apparent clinical symptoms were observed except infertility, subsequent studies were done to examine the effect of multiple doses of 10^8^cfu of *S. aureus* on histology of vagina and changes (if any) in the immunological markers.

### Cytokine Immunoassays

The cytokine levels were determined on days 0, 1, 2, 3, 4 and 5. In preliminary experiments, the levels of cytokines were estimated in vaginal secretions by taking vaginal lavages. However, as no significant changes were observed in both the estimated cytokines (data not shown), therefore, in order to reach conclusive evidence, vaginal homogenates from mice were subjected to cytokine analysis. In this case, the levels of IL-10 showed an increase on day 3 i.e. from 710.44±12.01pg/ml to 910.8±10.23pg/ml, but were normal thereafter (Figure 4A). However, no changes were observed in levels of TNF-α (Figure 4B).

### Histopathological Studies

Histopathological examination revealed no inflammatory changes in vagina on day 14 in the mice inoculated with 10^8^cfu of *S. aureus* for 10 days as compared to control group (Figure 5).

## Discussion

Bacterial interactions with spermatozoa lead to morphological defects as well as changes in functional parameters of spermatozoa *in vitro*. These may in turn lead to a decrease in the fertilization potential of sperm. Jiang and Lu [9] reported that *S. aureus* significantly immobilizes the spermatozoa *in vitro*. Villegas et al. [10] had further demonstrated that a single incubation with *Enterococcus fecalis*, *E. coli* and *S. aureus* induced apoptosis in human sperm. Though a lot of research has been done on the deleterious effects of bacteria on sperm *in vitro*, but still we have to go a long way in demonstrating these *in vivo* i.e. can these *in vitro* results be mirrored *in vivo*.

*S. aureus* is one of the dominant bacteria isolated from the semen samples of males complaining of infertility [2], [11]–[13]. But, the significance of this microbiological finding has been underestimated. To the best of our knowledge, no reports exist in literature which suggest its putative link with infertility. Therefore, the present study was undertaken to investigate the effect of intravaginal colonization with *S. aureus* on fertility in mice. The strain of *S. aureus* used in this study was isolated earlier in our laboratory from the cervix of a woman suffering from infertility. The strain showed notable spermagglutination property *in vitro* [7].

*In vivo* studies were aimed at establishing this strain in the vagina of Balb/c mice and to study its effect on reproductive potential. This strain was found to efficiently colonize the mouse vagina after repeated intravaginal inoculation of 10^4^, 10^6^ or 10^8^cfu for 10 consecutive days and became the only isolate in subsequent cultures. Mating of female mice on day 12 led to infertility in all the groups. However, when a non-spermagglutinating/immobilizing standard strain of *S. aureus* MTCC6625 was inoculated intravaginally even at a dose 10^8^cfu for 10 consecutive days, there was no effect on subsequent fertility as all the mice showed pregnancy and delivered pups.

Further, the duration of intravaginal inoculation was shortened to a single dose to achieve a practical model for the study. Even at a single dose the bacteria (10^6^ or 10^8^ cfu) were able to produce significant count in vagina except for 10^4^cfu which showed an early clearance by day 6. Mating was allowed in female mice both in the presence of organism and when the bacteria had cleared from the vagina. A notable observation was the correlation of infertility with the presence of spermagglutinating strain in the genital tract. This was evident from the result that female mice mated after clearance of the organism from the vagina or colonized with non-spermagglutinating standard strain showed fertility.

Repeated inoculations of 10^8^cfu for 10 days did not result in histopathological changes in vagina. However, any damage to the micro-structural integrity and subclinical inflammatory reactions in the vaginal epithelium may not be evident by simple histology (eosin/hematoxylin staining) [14]–[15]. Therefore, a non-invasive method to detect subtle tissue inflammation was a helpful adjunct to the histological findings. Proinflammatory cytokines (PICs) not only serve as sensitive markers for vaginal mucosal epithelial damage but also provide an insight into important biological changes that occur in the vagina [16]. For example, increased chemokine concentrations correlate with an influx of immune and inflammatory cells into the vaginal mucosa [16]–[19]. In the present study, we examined proinflammatory cytokine TNF-α and immuno-regulatory cytokines IL-10 as biomarkers and inflammatory mediators in the vaginal homogenates of mice and monitored in response to intravaginal inoculation of *S. aureus*. No significant changes were observed in the levels of TNF-α among the control and test groups, while a slight increase in the levels of IL-10 were observed on day 3 in the test group.

From the above results, it could be concluded that the presence of a spermagglutinating *S. aureus* strain (capable of producing an asymptomatic colonization i.e. without any overt symptoms in mouse reproductive tract) could induce infertility in female mice. As no histopathological changes were observed in the reproductive tract therefore infertility as a result of tubal blockage or any obstruction attributed to inflammation due to the presence of organism was ruled out. The results were further corroborated by establishing a non-agglutinating standard strain in the vagina which had no negative effects on subsequent fertility. However, further studies need to be carried out with more number of strains so as to obtain a statistically significant data and gather conclusive evidence in support of this hypothesis. Still, the study provides novel insights into not yet ventured aspect of infertility and suggests a relationship of bacteria with infertility.

## Supporting Information

Figure S1 **Spermagglutination observed 4h after mixing of human spermatozoa with** ***S. aureus*** **isolate (original magnification 400X).**
(JPG)

Figure S2 **No spermagglutination observed 4h after mixing of human spermatozoa with standard** ***S. aureus*** **MTCC6625 (original magnification 400X).**
(JPG)

Figure S3 **Standard curve for IL-10.**
(TIF)

Figure S4 **Standard curve for TNF-α.**
(TIF)
