# Supplementary figures and images for: Infertility as a Consequence of Spermagglutinating Staphylococcus aureus Colonization in Genital Tract of Female Mice
Source: PLoS One. 2012 Dec 18;7(12):e52325. doi: 10.1371/journal.pone.0052325 (PMC3525590; doi:10.1371/journal.pone.0052325)

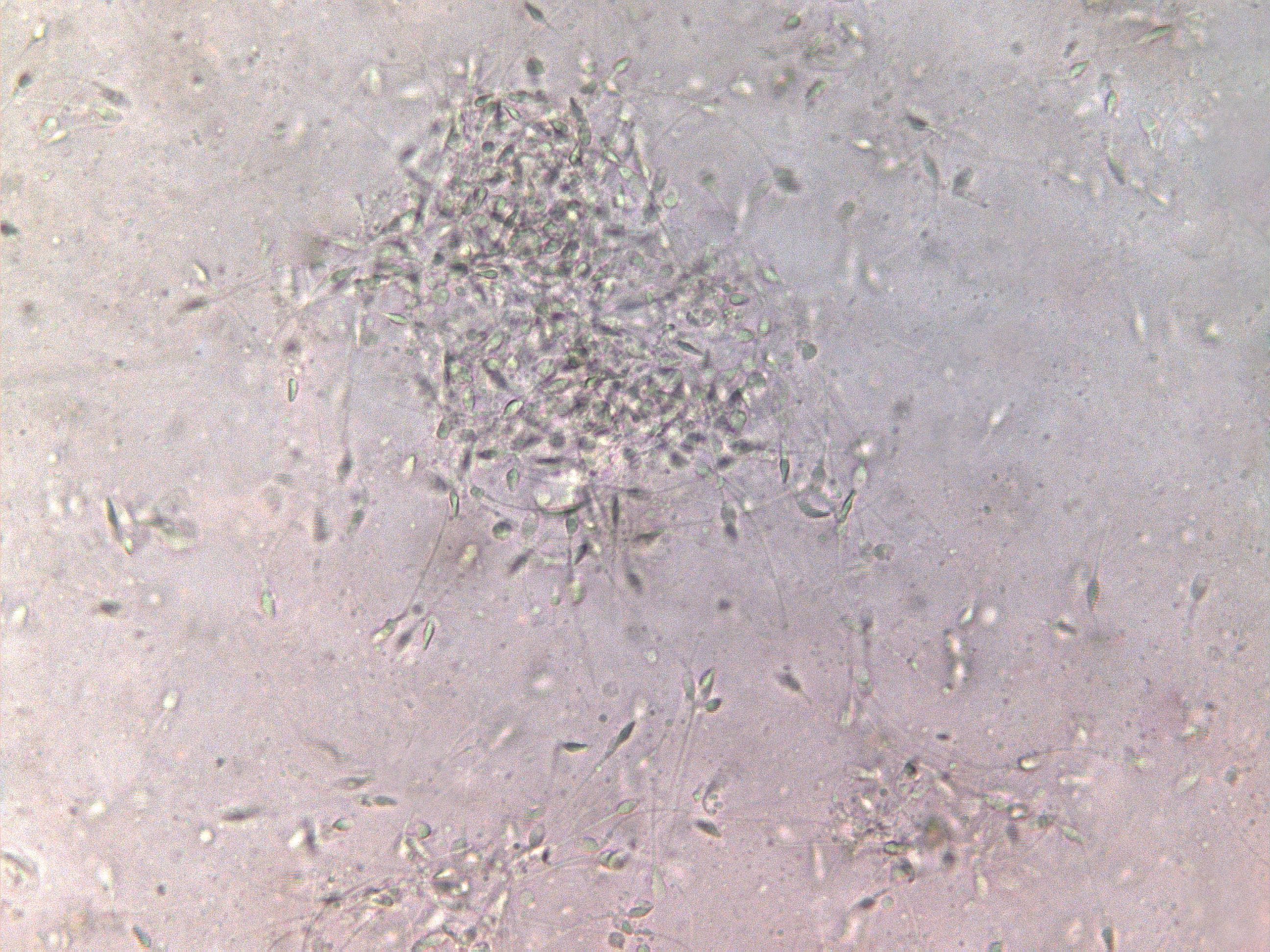

Supplement: Figure S1 — Spermagglutination observed 4h after mixing of human spermatozoa with S. aureus isolate (original magnification 400X). (JPG) [file pone.0052325.s001.jpg]

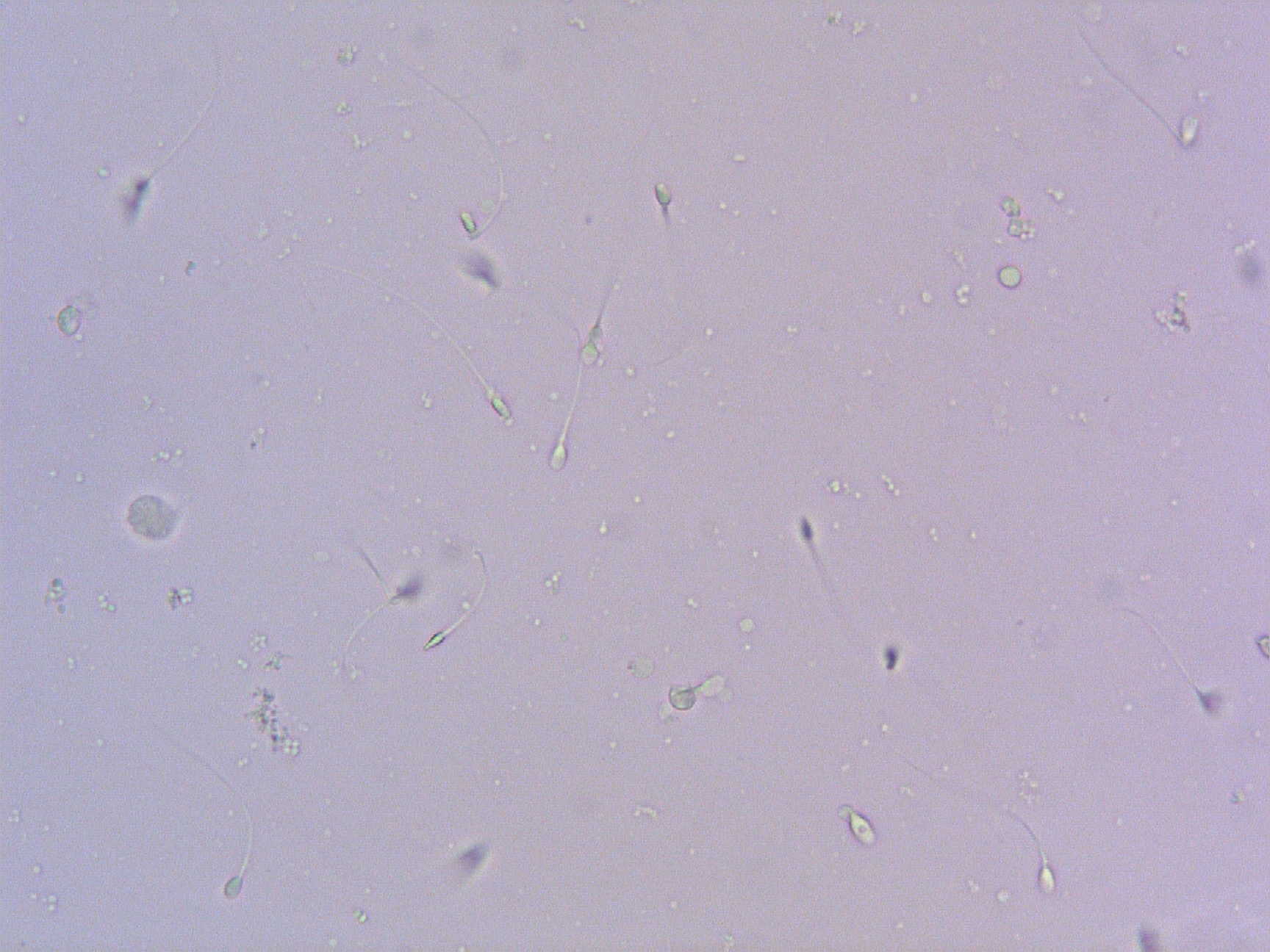

Supplement: Figure S2 — No spermagglutination observed 4h after mixing of human spermatozoa with standard S. aureus MTCC6625 (original magnification 400X). (JPG) [file pone.0052325.s002.jpg]

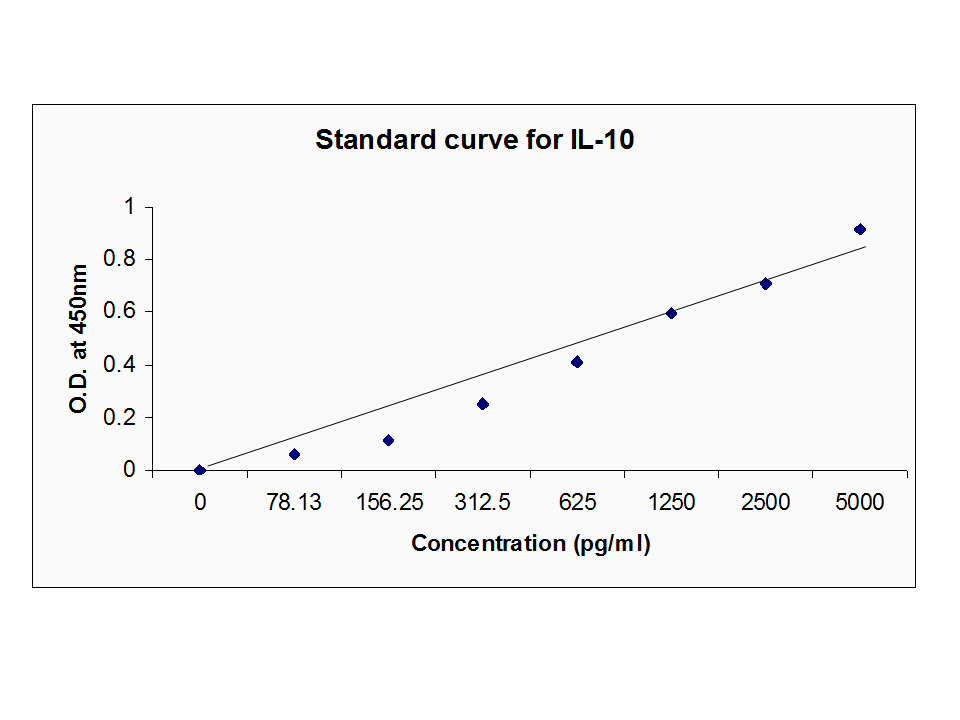

Supplement: Figure S3 — Standard curve for IL-10. (TIF) [file pone.0052325.s003.tif]

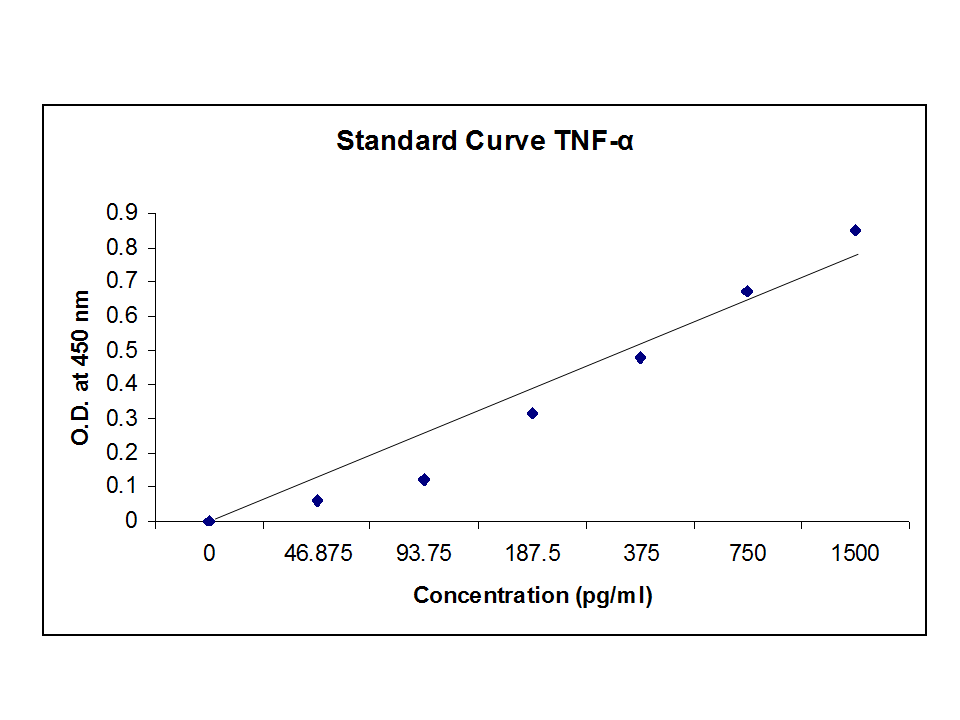

Supplement: Figure S4 — Standard curve for TNF-α. (TIF) [file pone.0052325.s004.tif]
